# Widespread Environmental Contamination with *Mycobacterium tuberculosis* Complex Revealed by a Molecular Detection Protocol

**DOI:** 10.1371/journal.pone.0142079

**Published:** 2015-11-11

**Authors:** Nuno Santos, Catarina Santos, Teresa Valente, Christian Gortázar, Virgílio Almeida, Margarida Correia-Neves

**Affiliations:** 1 Life and Health Sciences Research Institute (ICVS), School of Health Sciences, University of Minho, Braga, Portugal; 2 ICVS/3B’s, PT Government Associate Laboratory, Braga/Guimarães, Portugal; 3 Earth Sciences Institute (ICT), Pole of the University of Minho, Earth Sciences Department, University of Minho, Braga, Portugal; 4 SaBio (Health and Biotechnology), IREC, National Wildlife Research Institute (CSIC-UCLM-JCCM), Ciudad Real, Spain; 5 Centro de Investigação Interdisciplinar em Sanidade Animal (CIISA), Faculdade de Medicina Veterinária, Universidade de Lisboa (FMV-ULisboa), Lisboa, Portugal; University of Minnesota, UNITED STATES

## Abstract

Environmental contamination with *Mycobacterium tuberculosis* complex (MTC) has been considered crucial for bovine tuberculosis persistence in multi-host-pathogen systems. However, MTC contamination has been difficult to detect due to methodological issues. In an attempt to overcome this limitation we developed an improved protocol for the detection of MTC DNA. MTC DNA concentration was estimated by the Most Probable Number (MPN) method. Making use of this protocol we showed that MTC contamination is widespread in different types of environmental samples from the Iberian Peninsula, which supports indirect transmission as a contributing mechanism for the maintenance of bovine tuberculosis in this multi-host-pathogen system. The proportion of MTC DNA positive samples was higher in the bovine tuberculosis-infected than in presumed negative area (0.32 and 0.18, respectively). Detection varied with the type of environmental sample and was more frequent in sediment from dams and less frequent in water also from dams (0.22 and 0.05, respectively). The proportion of MTC-positive samples was significantly higher in spring (p<0.001), but MTC DNA concentration per sample was higher in autumn and lower in summer. The average MTC DNA concentration in positive samples was 0.82 MPN/g (CI_95_ 0.70–0.98 MPN/g). We were further able to amplify a DNA sequence specific of *Mycobacterium bovis/caprae* in 4 environmental samples from the bTB-infected area.

## Introduction

Bovine tuberculosis (bTB) is a zoonosis caused by *Mycobacterium bovis* or *Mycobacterium caprae*, both members of the *Mycobacterium tuberculosis* complex (MTC), whose natural hosts are wild and domestic mammals [1,2]. Bovine tuberculosis is a disease of economic and public health relevance subjected to eradication programs in livestock in many countries. As a consequence, bTB has been eradicated in a few countries but in others the disease persists despite massive investment in prevention, control and surveillance. This scenario has been attributed to the existence of wildlife reservoirs, such as possums (*Trichosurus vulpecula*) in New Zealand, Eurasian badgers (*Meles meles*) in the United Kingdom and Ireland and cervids in North America [3]. In several regions of Continental Europe, notably the Iberian Peninsula, bTB is maintained in a multi-host-pathogen system, with *M*. *bovis* and *M*. *caprae* circulating between sympatric wild ungulates (mostly wild boar *Sus scrofa* and red deer *Cervus elaphus*) and free-ranging domestic ungulates [2,4,5].

Transmission of *M*. *bovis* from an excretor to a susceptible host can occur by direct or indirect routes [6,7]. Direct transmission requires close contact between infected excretors and susceptible hosts [3]. Therefore, it is expected to play a major role in intraspecific transmission of infection, as close contact is common among individuals of the same species. However, close contact between individuals of different species seems to be rare [3,6,8,9] and so indirect routes are expected to play a crucial role in interspecific transmission. Indirect routes of transmission require the contamination of the environment with viable mycobacteria [6].

Indirect transmission of *M*. *bovis* was shown to occur scarcely in cattle grazing in either naturally or artificially infected pasture [7,10]. Nevertheless it is strongly suspected to play a major role in the white-tailed deer-cattle system of North America, where it has been experimentally shown to occur through contaminated feed [11,12,13]. It is also suspected to occur in other wildlife-cattle systems, such as badger-cattle in the United Kingdom and Ireland [6,8] and wild ungulates-cattle in the Iberian Peninsula [9]. In this later situation, environmental contamination of watering and feeding areas was proposed to be of epidemiological relevance [9].

Environmental contamination with MTC remains controversial and has not been thoroughly addressed in recent studies. Detection of *M*. *bovis* in soil samples has been reported to endure several weeks or months after inoculation, depending on the initial concentration used [6,10,14–16]. However, Young *et al*. [17] reported that mycobacterial DNA does not persist in the environment for more than 10 days outside a viable cell. Although it was experimentally shown that *M*. *bovis* DNA can persist in the environment for several months after no longer being recoverable by culture, this may reflect the lower sensitivity of bacteriological culture applied to environmental samples, when compared to molecular biology methods [17,18].

The lack of clear data on environmental contamination with MTC might be due mainly to the lack of sensitive and mass-scalable techniques to detect MTC in the environment [8,15,17,18]. Molecular techniques show a greater promise over bacteriological techniques to detect MTC in environmental samples [18]. Nevertheless, available protocols have exceedingly high detection limits, rendering them of limited usefulness as screening techniques, possibly due to the uneven distribution of mycobacteria in soil samples and the co-extraction of PCR inhibitors [19]. Young et al. [17] reported a protocol with detection limits of 10^2^−10^3^ cells/g soil, however, this protocol was not replicated by other research groups. Pontirolli et al. [20] optimized a protocol with a detection limit of 4.25 x 10^5^ cells/g soil, which is too high for the mycobacterial loads expected to occur in nature [18]. These two studies used direct extraction techniques, where DNA was extracted from an environmental sample, typically of 0.1–0.5 g. On the other hand, Sweeney et al. [21] described an immunomagnetic capture technique allowing the isolation and molecular detection of *M*. *bovis* from naturally contaminated soil samples. Despite this technical breakthrough in the study of the environmental contamination with pathogenic mycobacteria, this technique is difficult to scale up to test large numbers of samples and has not been replicated by other research groups.

In the present study we explore the real-life model of the multi-host pathogen system of Iberian Peninsula to assess the occurrence of environmental contamination with MTC at the interface between wild and domestic ungulates. The two central aims were: i) to define an improved protocol for the molecular detection and estimation of the concentration of MTC and *M*. *bovis* DNA in environmental samples, easy to scale-up and with higher sensitivity than previously published methods; ii) to apply this protocol to assess MTC environmental contamination in areas with well-described distinct bTB prevalence in wildlife.

## Material and Methods

### Study areas

Environmental samples (soil, sediment and water) were collected from two regions 70 km apart in southern Portugal, one known to be bTB-infected (geographical coordinates 4217747/673542 utm wgs84), where *M*. *bovis* or *M*. *caprae* have been isolated from the tissues of 42/60 hunted wild boar and 13/78 hunted red deer from 2009–2014; and another presumably bTB-free (geographical coordinates 4184462/615257 utm wgs84), where MTC have not been isolated from tissues of 84 wild boar and 3 red deer from 2009–2014. Wildlife bTB prevalence in these two areas was based on previously published data [22] and subsequent unpublished results. Both areas belong to the Mesomediterranean biogeographical region of the Iberian Peninsula [23], characterized by hot, dry summers and temperate humid winters, with a strongly seasonal pattern of precipitation. Landowners allowed the collection of the environmental samples from their properties. No other permissions were needed to collect soil, sediment and water samples. The study did not involve any endangered or protected species.

### Study design

**Fig 1:**
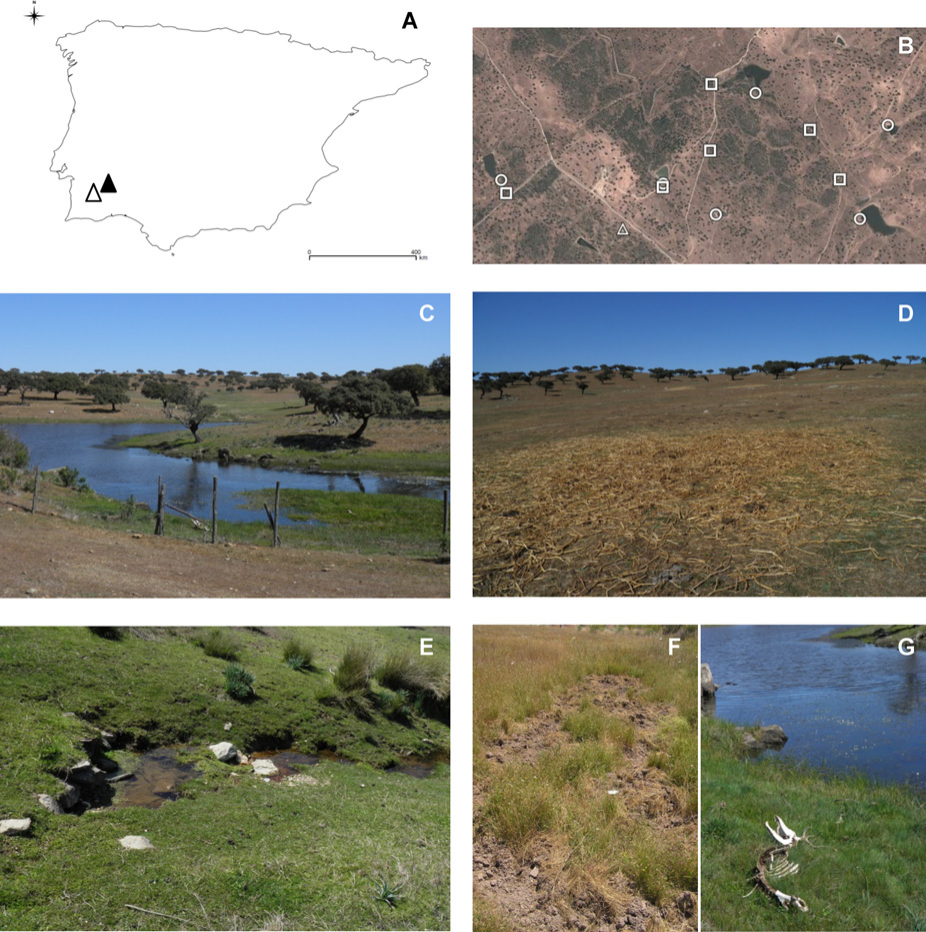
Aspects of the collection sites of environmental samples. (A) Map of the Iberian Peninsula highlighting the location of the bTB-positive (black triangle) and presumed bTB-negative (white triangle) study areas; (B) detail of sites in the bTB-infected area where samples were collected (squares: rivers, circles: dams; triangles: feeding sites); images of sample collection sites: (C) small dam, (D) feeding site, (E) seasonal river, (F) wild boar roots; (G) red deer skeleton besides a small dam.

Three types of *a priori* risk sites for the occurrence of environmental contamination with MTC were defined: i) small dams; ii) rivers (many seasonal) and iii) feeding areas (where hay or feed is provided, on the ground or in troughs, for cattle but also used by wild ungulates). Relevant aspects of the collection sites used for environmental samples are represented in Fig 1.

We collected a total of 319 environmental samples in the following subsets (Table 1): i) 71 sediment/water samples collected from dams in May and July 2013 and April 2014 in parallel at bTB-infected and presumed bTB-free study areas to compare the MTC DNA detection rates; ii) 204 samples from the bTB-infected study area, stratified by season (spring/summer/autumn/winter 2012) with the aim of describing the patterns of environmental contamination with MTC; iii) 44 samples opportunistically collected from soil rooted by wild boar, soil from vulture feeding stations and vulture feces.

**Table 1:** Samples collected and positive for MTC DNA by study area and sample type.

		Subsets of environmental samples		
Environmental samples		Patterns of MTC environmental contamination (no. positive/total tested)	Comparison between study areas (no. positive /total tested)	
		bTB-infected	bTB-infected	Presumed bTB-free
	Dams (sediment)	13/58	12/37	6/34
Standard	Dams (water)	3/57	n.a.	n.a.
samples	Rivers (sediment/water)	11/61	n.a.	n.a.
	Feeding areas (soil)	5/28	n.a.	n.a.
Opportunistic	Wild boar roots (soil)	4/16	n.a.	n.a.
samples	Vulture feeding stations (soil)	1/19	n.a.	n.a.
	Vulture feces (feces)	1/9	n.a.	n.a.
Total		38/248	12/37	6/34

Number of samples collected and number of samples positive for MTC DNA by study area and sample type. n.a.—not applicable.

### DNA extraction protocol

Environmental samples were collected in hermetic 1000 mlL polyethylene containers and kept refrigerated until analysis, which was performed 1–3 days post-collection. In order to homogenize the distribution of mycobacteria eventually present, on average 1,087±262 g (wet weight) of soil or sediment samples were soaked with a slight excess of distilled water in a 1,000 ml cylindrical container and agitated overnight at 150 rpm at 8°C in an incubator shaker (Multitron II, Infors AG, Bottmingen, Switzerland). After resting for 2 h at room temperature, 14 ml of the supernatant/sediment interface were collected and centrifuged at 2,566 g for 30 min, after which most of the supernatant was discarded and 0.5 ml aliquots of the sediment/supernatant interface collected for DNA extraction. 50 ml water samples were centrifuged at 2,566 g for 30 min, after which the extraction protocol was equal to soil and sediment samples.

DNA extraction was performed in triplicate for each environmental sample, using a slight modification of the protocol by Griffiths et al. [19]. Briefly, 0.5 ml of sample, 0.5 ml of 5% hexadecyltrimethylammonium bromide buffer and 0.25 ml phenol were added to a 2 ml screw-cap conical tube containing 100 μl of 0.1 mm zirconia/silica beads (Biospec Products, Bartlesville, USA). The mixture was subjected to 2 cycles of 30 s agitation at 5 m/s in a FastPrep 24 (MP Biomedicals, Santa Ana, USA), after which 0.25 ml chlorophorm were added and gently agitated for 60 s, followed by 5 min centrifugation at 16,627 g at 4°C. 500 μl of the aqueous phase was then extracted to a new tube and an equal volume of chlorophorm added, mixed by gentle agitation for 60 s and again centrifuged for 5 min at 16,627 g at 4°C. 300 μl of the aqueous phase were then extracted to a new tube and 400 μl of 30% polyethyleneglycol 6,000 solution in 1.6 M NaCl_2_ were added. The phase containing the precipitated DNA was collected and left to rest for 2 h at room temperature, followed by 10 min centrifugation at 19,283 g at 4°C. The supernatant was discarded and the pellet washed with 70% EtHO, centrifuged for 5 min at 16,627 g at 4°C, the supernatant again discarded and the pellet suspended in 50 μl of Tris-EDTA buffer. DNA was quantified and purity assessed using NanoDrop (ThermoScientific, Wilmington, USA). Negative controls for DNA extraction, consisting of 0.5 ml of water submitted to the same extraction protocol and interspersed with the environmental samples, were included at a rate of one for every 6 samples.

### Molecular detection

Every sample was subjected to a PCR targeting a 16SRNA sequence (1218–1432 bp sequence, depending on the microorganism [24]), common to all bacteria, as an inhibition external control. A modification of the protocol described by Hiraishi [25] was used, including the same set of primers described by this author (forward: 5’ AGAGTTTGATCCTGGCTCAG 3’, reverse: 5’ ACGGGCGGTGTGTACAAG 3’). Briefly, 250 ng DNA were added to a solution of 6.5 μl of NZYTech Green Master Mix (NZYTech, Lisbon, Portugal), containing 1.3 U Taq polymerase, 1.5 mM MgCl_2_, 1 μl of each primer at 10 mM and 5% dimethylsulfoxide, in a final volume of 25 μl. This mix was submitted to the following PCR cycles: initial denaturation at 93°C for 5 min, followed by 35 cycles of 93°C for 60 s, annealing at 55°C for 60 s and extension at 72°C for 60 s, with a final extension step of 72°C for 10 min. Inhibition was detected in 60/319 samples, which were then diluted 1:2 or 1:4 until inhibition disappeared. In all but 4 samples, PCR inhibition was avoided using this method; these 4 samples (1 water and 2 sediments from dams and 1 vulture feces) were removed from the analysis.

Previously to testing environmental samples we evaluated several conventional, nested and real-time PCR protocols, either previously published or developed in-house. The one showing the best performance was selected as screening protocol to detect MTC DNA in excretion routes from wild ungulates. As screening test for MTC DNA we selected a modification of the nested PCR protocol targeting a 110 bp sequence in IS6110 as described by Soo et al. [26], including the same set of primers described by those authors (external forward: 5’ CGTGAGGGCATCGAGGTGGC 3’, external reverse: 5’ GCGTAGGCGTCGGTGACAAA 3’, internal forward: 5’ CTCGTCCAGCGCCGCTTCGG 3’, internal reverse: 5’ GCGTCGGTGACAAAGGCCAC 3’). Briefly, 250 ng DNA were added to a solution of 7.5 μl of NZYTech Green Master Mix, containing 1.5 U Taq polymerase, 1.5 mM MgCl_2_, 1 μl of each primer at 10 mM and 5% dimethylsulfoxide, in a final volume of 25 μl. For the internal PCR 1 μl of the products of the external PCR was used as template. External PCR mix was submitted to the following PCR cycles: initial denaturation at 94°C for 5 min, followed by 26 cycles of 94°C for 30 s, annealing at 64°C for 15 s and extension at 72°C for 30 s, with a final extension step of 72°C for 3 min. Internal PCR mix was submitted to the same protocol, except that 30 cycles were used.

MTC-positive samples were submitted in triplicate to a hemi-nested PCR protocol specific for *M*. *bovis/caprae*, targeting a 306 bp sequence of RD12 (external and internal forward: 5’ AGCAGGAGCGGTTGGATATTC 3’, external reverse: 5’ CGCCTACGCGTACTGGTATT 3’, internal reverse: 5’ GTGTTGCGGGAATTACTCGG 3’). The internal and external forward primers were previously described [27], while the external reverse primer was designed *in silico* using the software Primer-Blast [24]. Briefly, 250 ng DNA were added to a solution of 7.5 μl of NZYTech Green Master Mix, containing 1.5 U Taq polymerase, 2.5 mM MgCl_2_, 1 μl of each primer at 10 mM and 5% dimethylsulfoxide, in a final volume of 25 μl. In the internal PCR, 1 μl of the products of the external PCR was used as template. External PCR mix was submitted to the following PCR cycles: initial denaturation at 95°C for 5 min, followed by 35 cycles of 95°C for 30 s, annealing at 56°C for 30 s and extension at 72°C for 30 s, with a final extension step of 72°C for 5 min. Internal PCR mix was submitted to the same protocol, except that 45 cycles were used.

MTC-positive samples were also submitted in triplicate to another hemi-nested PCR that allows for the differentiation of *M*. *microti*, *M*. *tuberculosis*, *M*. *africanum* and *M*. *pinnipedii* from other members of the MTC, targeting a 369 bp sequence of RD12 (forward: 5’ AGCAGGAGCGGTTGGATATTC 3’, external reverse: 5’ CGATCGCCGTGATCACAAAC 3’, internal reverse: 5’ GGGAGCCCAGCATTTACCTC 3’). The internal and external forward primers were previously described [27], while the external reverse primer was designed *in silico* using the software Primer-Blast [24]. Briefly, 250 ng DNA were added to a solution of 7.5 μl of NZYTech Green Master Mix, containing 1.5 U Taq polymerase, 2.5 mM MgCl_2_, 1 μl of each primer at 10 mM and 5% dimethylsulfoxide, in a final volume of 25 μl. In the second (internal) PCR, 1 μl of the products of the first (external) PCR was used as template. External PCR mix was submitted to the following PCR cycles: initial denaturation at 94°C for 5 min, followed by 35 cycles of 94°C for 30 s, annealing at 58°C for 30 s and extension at 72°C for 30 s, with a final extension step of 72°C for 3 min. Internal PCR mix was submitted to the same protocol, except that we used 45 cycles, 65°C annealing temperature and 2 mM MgCl_2_ were used.

**Fig 2:**
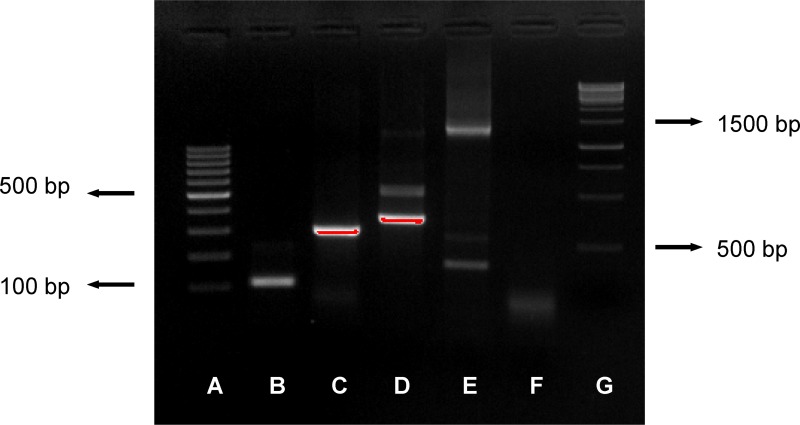
Image of gel showing all bands obtained with the present protocol. Image of gel showing amplification of the PCR protocols described: (A) GeneRuler 100bp DNA ladder; (B) MTC DNA IS6110 (110 bp); (C) *M*. *bovis*/*caprae* DNA RD12 (306 bp); (D) *M*. *microti/tuberculosis/ africanum/pinnipedii* DNA RD12 (369 bp); (E) 16SRNA (1218–1432 bp); (F) negative control; (G) GeneRuler 1kbp.

PCR products were visualized by electrophoresis in 2% agarose gel with GreenSafe Premium (NZYTech, Lisbon, Portugal) and GeneRuler 100 bp DNA Ladder (Thermo Fisher Scientific, Walthman, Massachusetts, USA) and photographed under UV light with Alpha Imager (ProteinSimple, San Jose, California, USA) (Fig 2). The preparation of the nested PCR master mixes took place in a room where no other work with MTC took place and physically separated from the rooms where the addition of the DNA templates was performed. Negative controls for the PCR protocol, consisting of 1 μl sterile water instead of extracted DNA, were included in all PCR runs at a rate of one for every 6 samples. This means that every PCR run included 1 negative control (either PCR or DNA extraction control) interspersed with every 3 samples (S1 Dataset). Negative controls were handled as the samples to be tested. The specificity of the PCR assays is supported in the literature [26,27] and was confirmed by *in silico* analysis and tested in an assay including *M*. *tuberculosis*, *M*. *bovis* BCG, *M*. *bovis* and *M*. *caprae* field isolates, *M*. *avium*, *M*. *smegmatis* and *E*. *coli*.

### Limits of detection

Soil samples were collected from peri-urban soils in Braga, Portugal where no MTC contamination was expected to occur, while water from ponds from the same region was used for bacterial suspensions. Ten replicates of whole-community DNA extraction and nested PCR assays were performed to assure these substrates were free from detectable levels of MTC contamination. Soil and water samples were seeded with 2-fold decreasing concentrations of *M*. *bovis* Bacillus Calmette-Guérin (BCG) strain Pasteur, determined by colony-forming units (CFU). Negative controls were included in each assay, consisting of the same substrate (soil or water) inoculated with the same volume of sterile water. After seeding, the samples were manually agitated to homogenize the mycobacterial distribution and subjected to the molecular detection techniques previously described. The 100% limit of detection (LD100) and 50% limit of detection (LD50) were determined based on the results of 7 molecular detection assays in the seeded samples. In order to assess the effect of increasing sample volume in the efficiency of the MTC detection in environmental samples, 0.5, 5, 50 and 500 g of soil were inoculated with 10^6^ CFU/g of BCG and subjected to the previously described DNA extraction and PCR amplification protocols.

### Most Probable Number

MTC DNA concentration was estimated by the Most Probable Number (MPN) [28] based on positive/negative nested PCR data on serial dilutions of DNA. Briefly, serial 10 fold dilutions of MTC-positive DNA samples were submitted to multiple nested PCR protocols as previously described. Undiluted DNA was assayed 3–8 times, 1:10 DNA 3–6 times and 1:10^2^ DNA 1–2 times until one dilution yielded at least two negative results. The dilution at which no amplification begins to occur indicates that the DNA has been diluted so much as to be absent and is used to estimate the original concentration. The software MPN Calculator Build 23 (http://www.i2workout.com/mcuriale/mpn/) was used to calculate the MPN MTC DNA concentration in environmental samples.

### Meteorological data

Meteorological data were obtained from IPMA [29] concerning the weather station located at Beja (geographical coordinates 593635/4215076 utm wgs84), 33 and 75 km from the study areas. Data consisted of air temperature and humidity (average, minimum, maximum), wind speed, soil temperatures (grass, 5 cm, 10 cm), soil water content, evapotranspiration (ET0, Penman-Monteith, model Aladin, FAO method), global solar radiation, precipitation and number of days with fog or rain. Overall the second half of 2012 was characterized by heavy rainfall, after 2011 and the first half of 2012 being very dry, with extreme drought in both study areas and over much of Iberian Peninsula [29]. A Principal Components Analysis was performed in order to highlight which meteorological variables are more strongly related to the probability of detecting MTC DNA in the environment.

### Physical-chemical characterization of the samples

After collection, samples were immediately refrigerated, transported in polyethylene bottles and stored in the dark at 4°C until analysis. A subset of sediments or soil samples (n = 7) were dried at 40°C for 72 h and the organic matter content was estimated by loss on ignition method. Quantitative assessment of percentage for different grain sizes in the coarser fractions was performed by screening, using a standard series of sieves between 0.062 and 2 mm. Silt- and clay-sized material classification was obtained using automated SediGraph 5100 (Micromeritics, Norcross, USA). The texture classification was based on the United States Department of Agriculture soil texture diagram [30].

In a subset of water samples (n = 12) pH and electric conductivity were measured with multiparameter Crison MM40+ (Crison Instruments, Barcelona, Spain). Before use, electrodes were calibrated and/or tested for accuracy, according to the manufacturer's instructions. Laboratory analyses were performed for anions by ion chromatography with suppressed conductivity detection (761 Compact IC, Metrohm AG, Herisau, Switzerland) and for alkalinity by volumetric determination [31].

### Statistical analysis

Principal Components Analysis and Pearson’s χ^2^ were performed using IBM SPSS Statistics (SPSS, Chicago, Illinois, USA); graphics were produced in Excel 2007 (Microsoft, Redmond, Washington, USA); and confidence intervals for the positivity rates were calculated using VassarStats (http://vassarstats.net/).

## Results

### Limits of detection depend on the type and amount of substrate

As PCR results have been shown to be influenced by characteristics of the substrate we considered of relevance to perform physical and chemical characterization of the soil and water used in this study. Overall the soil and sediment samples analyzed were of sandy loam texture and with low clay content, while the water showed neutral pH and low total dissolved solids (Table 2).

**Table 2:** Physical-chemical parameters of the environmental samples.

Type of sample	Analytical parameter	Avg ± SD
	Texture	Sandy loam
Soil/sediment	Sand (%)	56.3 ± 26.5
(n = 7)	Silt (%)	36.4 ± 22.0
	Clay (%)	7.3 ± 4.7
	Organic matter (%)	5.8 ± 4.3
	pH	6.9 ± 0.16
	Electric conductivity (μS/cm)	108.1 ± 38.7
	Total Dissolved Solids (mg/l)	69.2 ± 24.9
	Total alkalinity (mg/l CaCO_3_)	37.2 ± 11.9
Water	Fluoride (mg/l F^-^)	0.077 ± 0.044
(n = 12)	Chloride (mg/l Cl^-^)	17.717 ± 7.220
	Nitrite (mg/l NO_2_^-^)	0.064 ± 0.070
	Nitrate (mg/l NO_3_^-^)	13.839 ± 14.144
	Phosphate (mg/l PO_4_^3-^)	0.548 ± 0.816
	Sulphate (mg/l SO_4_^2-^)	6.655 ± 3.274

Physical-chemical characteristics of the environmental samples analyzed.

The determination of the MTC DNA detection limit, using BCG inoculation, revealed that it varies between soil and water. We observed that in soil both LD100 and LD50 were 4 x 10^4^ CFU/g while in water the LD100 was 5 x 10^5^ CFU/ml and the LD50 was 10^5^ CFU/ml. Regarding the *M*. *bovis*/*caprae* molecular detection protocol in water the LD100 was 5 x 10^5^ CFU/ml and the LD50 was 10^5^ CFU/ml, while in soil the LD100 was 10^6^ CFU/g and the LD50 was 4 x 10^4^ CFU/g.

Interestingly we observed that the initial volume of the sample had an impact on the detectability of MTC DNA, as 500 g of soil inoculated with 10^6^ CFU/g BCG yielded 100% positive results with an estimated concentration of 42 MPN/g (CI_95_ 13–130 MPN/g), while 50 g of soil inoculated with the same concentration of BCG yielded 80% positive results with an estimated concentration of 1.9 MPN/g (CI_95_ 0.7–5.2 MPN/g). No positive results were obtained for 5 g and 0.5 g of soil inoculated with the same concentration of BCG. Furthermore, samples of 500 g of soil inoculated with 10^5^ CFU/g BCG yielded a concentration of 1.9 MPN/g (CI_95_ 1.1–3.5 MPN/g) in the sediment, 0.8 MPN/g (CI_95_ 0.4–1.1 MPN/g) in the sediment/supernatant interface and no detection in the supernatant.

### Environmental MTCcontamination was detected in all types of samples

The proportion of MTC-positive samples in the bTB-infected area was higher (0.32, CI_95_ 0.20–0.49) than in the bTB presumed negative area (0.18, CI_95_ 0.08–0.34), although this difference did not reach statistical significance (p = 0.15, Pearson’s χ^2^) (Table 1).

From the bTB-infected area, 38/248 (0.15, CI_95_ 0.11–0.20) environmental samples were positive for MTC DNA (Table 1). MTC DNA was detected more often in sediment from dams (0.22, CI_95_ 0.14–0.35), in mixed sediment/water from rivers (0.18, CI_95_ 0.10–0.29) and soil from feeding points (0.18, CI_95_ 0.08–0.36) and significantly less in water from dams (0.05, CI_95_ 0.02–0.14) (p = 0.05, Pearson’s χ^2^). In the opportunistically collected samples MTC DNA was detected in 4/16 wild boar roots, 1/9 vulture feces and 1/19 soil from vulture feeding stations (Table 1).

In 4 environmental samples from the bTB-infected area the *M*. *bovis*/*caprae*-specific sequence was amplified being two sediments from dams, one from a river and one from a feeding site. Seven samples were positive for the *M*. *microti*/*tuberculosis*/*africanum*/*pinnipedii*-specific sequence, all from the bTB-infected study area and spanning every type of environmental sample analyzed (Table 3).

**Table 3:** Environmental samples from which *M*. *bovis/caprae*- or *M*. *microti/tuberculosis/africanum/pinnipedii*-specific sequences were amplified.

Mycobacterial species	Type of sample	Date of collection	MTC DNA estimated concentration (MPN/g)	CI_95_ MTC DNA estimated concentration (MPN/g)
	Feeding site (soil)	March 2012	0.62	0.23–1.7
*M*. *bovis*/*caprae*	River (sediment/water)	March 2012	0.93	0.34–2.5
	Dam (sediment)	March 2012	1.8	0.73–4.3
	Dam (sediment)	May 2012	0.45	0.14–1.4
	River (sediment/water)	May 2012	2.6	0.83–8.4
	Dam (sediment)	December 2012	0.93	0.23–3.8
*M*. *microti*/*tuberculosis*/ *africanum*/*pinnipedii*	Feeding site (soil)	January 2013	0.26	0.04–1.9
	Dam (water)	January 2013	1.2	0.37–3.8
	Dam (sediment)	January 2013	0.26	0.07–1.1
	Dam (sediment)	May 2013	2.8	0.96–8.4
	Dam (sediment)	April 2014	39.0	15.0–100.0

Details of the environmental samples from which the *M*. *bovis/caprae*-specific or the *M*. *microti/tuberculosis/africanum /pinnipedii*-specific sequences were amplified.

### Environmental MTCcontamination was detected mostly in spring

The proportion of positive samples for MTC DNA was significantly higher in spring than in the other seasons (p<0.001, Pearson’s χ^2^) (Table 4). This overall seasonal pattern was replicated in sediment samples from dams, in mixed sediment/water from rivers and soil from feeding points. In feeding points no MTC DNA was detected neither in summer nor in autumn, while in water from dams one positive result was obtained every season except in autumn (Table 4). The proportion of MTC DNA positive samples in sediment from dams was not significantly different when comparing the spring of 2012 (8/15 positive samples), 2013 (4/12) and 2014 (6/15) (p = 0.56, Pearson’s χ^2^).

**Table 4:** Proportion of environmental samples where MTC DNA was amplified in the bTB-infected area by sample type and season.

Environmental sample	Spring		Summer		Autumn		Winter	
	no.	Proportion positive (CI_95_)	no.	Proportion positive (CI_95_)	no.	Proportion positive (CI_95_)	no.	Proportion positive (CI_95_)
Dam (sediment)	14	0.57 (0.33–0.79)**	15	0.07 (0.01–0.31)	15	0.13 (0.04–0.38)	14	0.14 (0.04–0.40)
Dam (water)	14	0.07 (0.01–0.31)	14	0.07 (0.01–0.31)	15	0.00 (0.00–0.20)	14	0.07 (0.01–0.31)
River	15	0.47 (0.25–0.70)**	15	0.13 (0.04–0.38)	16	0.13 (0.04–0.36)	15	0.00 (0.00–0.20)
Feeding site	7	0.57 (0.25–0.84)*	8	0.00 (0.00–0.32)	7	0.00 (0.00–0.35)	6	0.17 (0.03–0.56)
Total	53	0.40 (0.28–0.54)***	52	0.08 (0.03–0.18)	53	0.08 (0.03–0.18)	49	0.08 (0.03–0.19)

Proportion of samples with MTC DNA amplification in the bTB-infected area by sample type and season, with confidence intervals and statistically significant differences between seasons highlighted (Pearson’s χ^2^).

* p<0.05

** p<0.01

***p<0.001

### Estimated MTC DNA concentration showed a bimodal distribution

**Fig 3:**
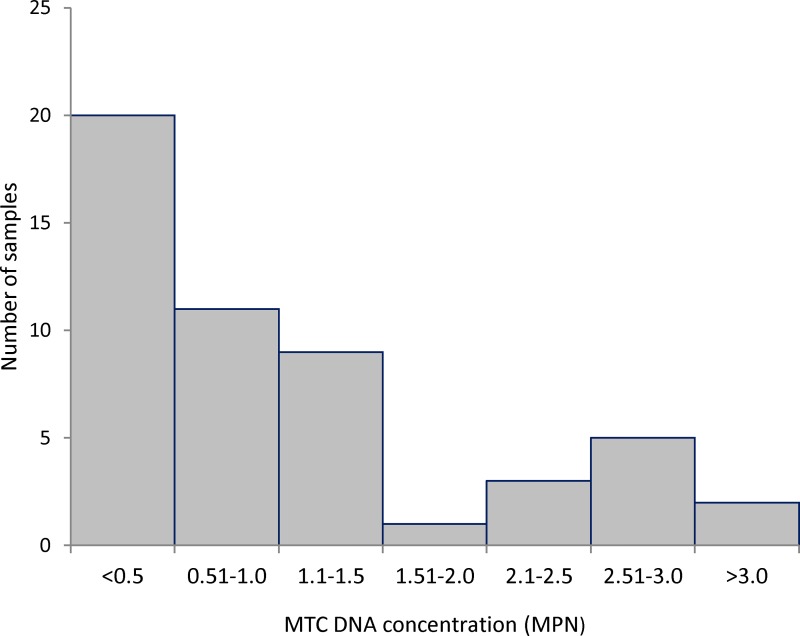
Distribution of MTC DNA concentrations. Histogram of MTC DNA concentrations estimated by the Most Probable Number method in the environmental samples from which MTC DNA was amplified (n = 56), both study areas combined.

On the 56 samples positive for MTC DNA the average concentration was 0.82 MPN/g (CI_95_ 0.70–0.98 MPN/g). The highest concentration recorded was 39 MPN/g (CI_95_ 15–100 MPN/g), in a sediment/water sample collected from a dam in the bTB-infected area. The distribution of the MTC DNA concentrations followed a bimodal pattern with two modes in the classes <0.5 MPN/g and 2.51–3.0 MPN/g (Fig 3).

**Fig 4:**
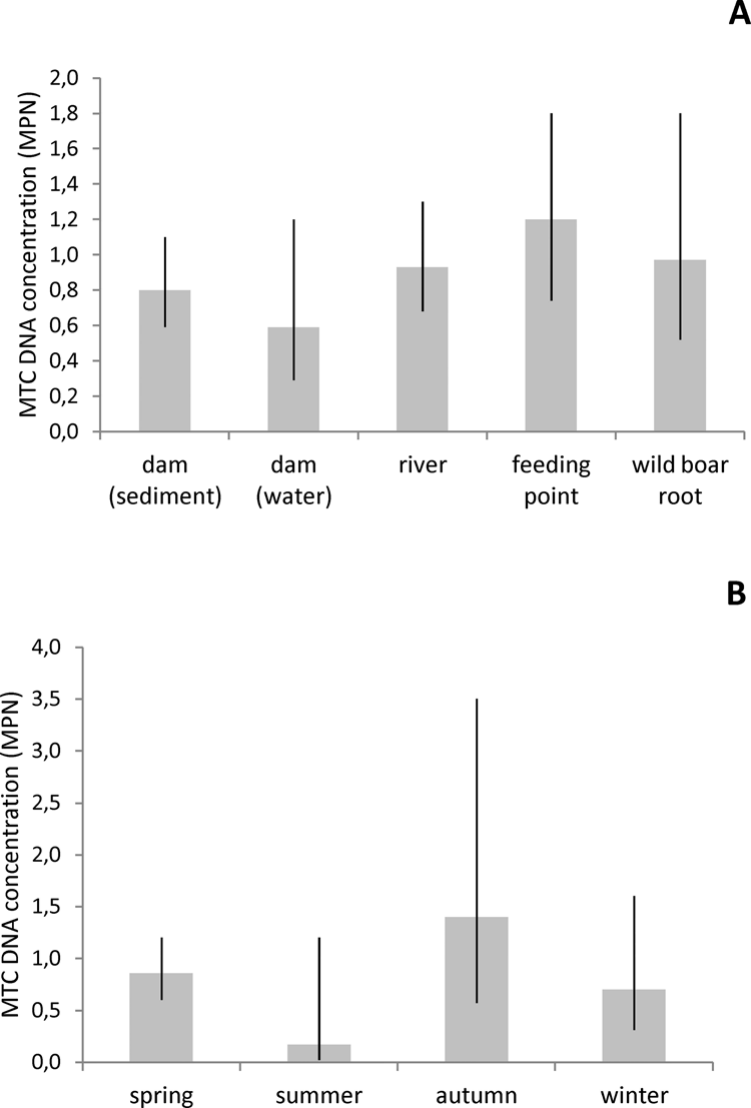
Estimated MTC DNA concentrations by season and sample type. Average MTC DNA concentration estimated by the Most Probable Number in environmental samples from the bTB-infected area, by sample type (A) and season (B), with 95% confidence intervals.

MTC DNA concentration was not significantly different across sample types, although a tendency was seen for higher concentration in feeding points and lower in water from dams (Fig 4A). Also, MTC DNA concentration tended to be higher in samples collected during autumn and lower in summer (Fig 4B), although no statistically significant influence of season was found.

### Meteorological variables associated with the probability of MTC DNA detection

A Principal Components Analysis showed that temperature (air, soil 5 cm and soil 10 cm) and evapotranspiration are the variables most consistently positively associated with the probability of detection of MTC DNA in environmental samples when considering as time range for the meteorological data the 6 months, 1 month or 1 week previous to the collection of the samples (Table 5).

**Table 5:** Meteorological variables with the highest loadings on the Principal Components Analysis.

Meteorological variables	Previous 6 months	Previous 1 month	Previous 1 week
Air temperature (average)	0.982	0.985	0.938
Soil temperature (grass)	0.962	n.a.	n.a.
Soil temperature (depth 5 cm)	0.987	0.979	0.954
Soil temperature (depth 10 cm)	0.983	0.979	0.956
Evapotranspiration	0.977	0.996	0.957
Water content in soil	-0.990	n.a.	n.a.
Solar radiation	0.926	n.a.	0.953
Wind speed (average)	0.980	n.a.	n.a.
Fog-days	-0.989	n.a.	n.a.
Precipitation	0.900	0.947	n.a.
Rain-days		0.943	n.a.
Variation explained, 2 components combined	0.956	0.943	0.885

Meteorological variables with the highest loadings on the first two components of the Principal Components Analysis, with detection of MTC DNA as dependent variable. n.a.—not applicable.

## Discussion

Contamination of the environment with *M*. *bovis/caprae* is considered an important contribution to the persistence and interspecific spread of bTB, nevertheless methodological issues have impaired our knowledge on this matter [7,32]. The present study describes and applies an improved protocol for the molecular detection of MTC in environmental samples and reports for the first time the widespread occurrence of MTC DNA in the environment in areas where bTB is highly prevalent in wildlife. This contamination is detected in all types of *a priori* defined risk sites, where wild and domestic ungulates assemble, such as feeding and watering places. Spatial aggregation of wildlife at feeding or watering points was previously shown to be a risk factor for bTB prevalence [5]. Nevertheless, interspecific direct contact seems to be rare because of temporal segregation in their use [9]. Indirect transmission of bTB through environmental contamination with MTC provides a means to explain this risk effect, however mechanisms of infection from environmental sources still remain to be explained. In cattle, soil consumption when feeding in contaminated pasture has been proposed as a mechanism by which infection may occur [6]. Red deer have a mixed grazer and browser diet [33] which could theoretically put them at lower risk of infection thorough feeding. On the other hand, wild boar consistently root through soil when feeding [34] and so could be more exposed. Interestingly, MTC was detected in 4/16 wild boar roots, in an area where bTB prevalence in this species is 0.70 (unpublished data). Wild boar usually shows bTB prevalence much higher than sympatric red deer and their necrophagy habits have been proposed as a means to explain this difference [22]. Given the widespread environmental contamination we detected, their fossatorial habits could further explain this apparent increased exposure to infection.

The protocol we describe has the novelty of starting from a large volume of soil and sediment substrate, which we show to improve the detection rate. In fact, most published studies extract DNA from small volumes of substrate (0.1–1.0 g) [17,20,21]; by incorporating a homogenization step through the overnight agitation of approximately 1,000 g of substrate the detection rate increases considerably. We hypothesize that the agitation of the substrate in water homogenizes the MTC distribution in the substrate and so improves the detectability. In fact it was speculated that the uneven distribution of MTC in environmental samples hampers their molecular detection, together with the co-extraction of PCR inhibitors [20]. In our study inhibition was detected in 18.8% of the environmental samples but could be managed by the dilution of the samples up to 1:4 in all but 1.3% of them. PCR inhibitors such as humic compounds concentrate in the organic matter [20,35], the content of which was average to high in our samples. Also clay adsorbs DNA, hampering its extraction from soil samples [17], nevertheless clay content was low in the environmental samples analyzed in the present study (Table 2).

The LD100 of the MTC molecular detection protocol we describe is approximately 10 times lower than the one reported by Pontirolli et al. [20] for soil samples. Our protocol detects MTC DNA in sediment from dams in the bTB high-prevalence study area at a rate almost double than that of an area where bTB has not been detected in wild and domestic ungulates despite active surveillance. Although the difference is not statistically significant, this suggests that environmental contamination with MTC is higher in areas where bTB is highly prevalent in wild ungulate populations. The low success in the specific identification precluded any conclusion on the MTC species responsible for the positive results from the presumed bTB-free study area, which could be caused by environmental contamination with MTC other than *M*. *bovis*/*caprae*.

In fact, although our protocol represents a clear improvement from the previously published, it has limitations, the first of which is the low success rate in the specific identification of MTC. MTC includes several species, namely *M*. *tuberculosis*, *M*. *canettii*, *M*. *africanum*, *M*. *bovis*, *M*. *caprae*, *M*. *microti* and *M*. *pinnipedii* [1,32]. The first three species are not known to have other maintenance host besides humans [1] and so are very unlikely to be widespread in the environment in semi-natural areas with low human density and low human TB prevalence such as our study areas. *M*. *pinnipedii* natural hosts are marine mammals [36] and so is also unlikely to be present in environmental samples from our study areas. On the other hand *M*. *bovis* and *M*. *caprae* are the etiological agents of bTB and have been isolated in wild and domestic hosts in our high-prevalence study area [4,22]. DNA from these two mycobacterial species was detected in 4 samples and they could account for a larger proportion of the MTC detected in environmental samples. Nevertheless, the 25 x higher LD100 of the *M*. *bovis/caprae*-specific molecular detection protocol in soil samples compared with the MTC molecular detection protocol precluded estimating their proportion in our sample. Although *M*. *microti* has not been reported in wildlife in the Iberian Peninsula, its natural hosts are rodents [37] and could plausibly be present in our study areas and account for an unknown proportion of the MTC DNA positive results from both study areas, but further work is needed on this subject.

MTC DNA concentrations in the environment follow a bi-modal pattern of two distributions roughly separated at 2 MPN/g (Fig 3). A possible explanation is that the lowest concentrations of MTC DNA could originate from standard excretion from infected animals, while the highest concentrations could come from occasional events leading to higher focal contamination, such as the location of carcasses of infected animals (Fig 1G) or mycobacterial excretion by “super-shedder” hosts, such as described for the badger [38]. Further work is needed to explain this result.

MTC presence in the environment is dependent on excretion rates from infected animals and also on the survival of mycobacteria. MTC DNA detection rates are significantly higher in spring in all types of samples except water from dams. The fact that no significant differences in detection rates are found between three consecutive springs suggests that this is a consistent seasonal phenomenon. In fact spring in areas of the Iberian Peninsula with Mediterranean climate is characterized by moderate air and soil temperatures (average 15.8 and 16.6°C respectively, spring 2012) and relatively high water content of soil (average water content of soil 49.8%, spring 2012) [29]. In our study, MTC DNA detection in environmental samples was positively associated with air and soil temperatures and evapotranspiration. *M*. *bovis* survival in the environment was shown to be influenced by meteorological determinants; Fine et al. [15] reported that temperature (only air temperature was measured in that study) was significantly and positively associated with *M*. *bovis* persistence in the environment in Michigan. In the present study, the lowest average MTC concentration is found in summer, when climatic conditions are theoretically the worst for mycobacterial survival because of extremely high temperature (average maximum air temperature 32.6°C, average soil temperature 10 cm 26.4°C, summer 2012) and low water content of soil (average 2.0%, summer 2012) [15]. Soil dryness was expected to be an important limiting factor for MTC survival in feeding areas, where the only water content of soil is that of rainfall. In fact, it is noteworthy that no MTC DNA is detected in soil samples from feeding areas collected during summer, down from 0.57 positivity rate in the previous spring. In our study, MTC DNA detection rates and concentration are not significantly different between substrates (soil, sediment and water) as also reported by Fine et al. [15].

Summarizing, we describe an improved version of a protocol for the sensitive detection of MTC DNA that is simple, mass-scalable and applicable in several substrates of environmental samples. This protocol allowed for the first time the detection and description of overall spatio-temporal patterns of environmental contamination with MTC in areas where bTB is highly prevalent in wild ungulates. The data generated raises several questions which will need further study, such as the specific identification of MTC involved, assessment of its viability, quantification of the contribution of indirect transmission on bTB persistence in multi-host-pathogen systems and investigation of MTC excretion from infected hosts.

## Supporting Information

S1 Dataset MTC nested PCR dataset. Dates and results of the nested PCR protocols targeting IS6110 and RD12, including the negative controls. In brackets the initial date the nested PCR was performed, which was repeated whenever the negative control amplified a sequence.
(XLSX)
